# Draft Genome Sequence of *Bacillus* sp. Strain 007/AIA-02/001, Isolated from the Marine Sponge *Coelocarteria singaporensis*

**DOI:** 10.1128/MRA.00389-19

**Published:** 2019-08-29

**Authors:** Ji Fa Marshall Ong, Hui Chin Goh, Lik Tong Tan

**Affiliations:** a Natural Sciences and Science Education, National Institute of Education, Nanyang Technological University, Singapore; b p53 Laboratory, Agency for Science, Technology and Research (A*STAR), Singapore; University of Maryland School of Medicine

## Abstract

We report the draft genome sequence of a marine bacterium, *Bacillus* sp. strain 007/AIA-02/001, isolated from the marine sponge Coelocarteria singaporensis, obtained from water off the coast of Singapore. The analysis of the bacterial genome using the bioinformatics tool antiSMASH 4.0.2 showed the presence of a number of unique natural product biosynthetic pathways.

## ANNOUNCEMENT

Microbes isolated from marine-derived samples, such as marine invertebrates and sediments, are known to produce structurally novel bioactive compounds with significant therapeutic activities, such as anticancer, antifungal, antibiotic, and quorum sensing inhibitory properties (1–5). As such, the marine habitats are a tremendous resource for the discovery of novel drug leads in drug discovery and development efforts (6). Preliminary investigation carried out at our laboratory has revealed that there are potential quorum sensing inhibitory molecules produced by marine bacterial strains associated with deep-water marine sponges collected in the Singapore Strait (5). We sequenced the genome of a representative *Bacillus* sp. strain, 007/AIA-02/001, isolated from samples of the marine sponge Coelocarteria singaporensis, collected from the seabed surface using a rectangular dredge in the Singapore Strait (1.173183N, 103.762150E) on 14 February 2017.

Homogenate from the sponge sample was processed in sterile artificial seawater, plated onto Sigma-Aldrich *Actinomycetes* isolation agar, and incubated at 25°C for 13 days before the bacterial colony was isolated and purified. Amplification of the 16S rRNA with the universal primers 27F (5′-GAGTTTGATCCTGGCTCAG-3′) and 1525R (5′-33 AGAAAGGAGGTGATCCAGCC-3′) and sequencing confirmed that isolate 007/AIA-02/001 is a member of the *Bacillus* genus.

Genomic DNA was obtained from the culture of strain 007/AIA-02/001 and incubated at 25°C for 2 days in Difco marine agar. The genomic DNA (gDNA) was isolated using a Quick-DNA fungal/bacterial microprep kit (Zymo Research), followed by ethanol precipitation in order to obtain good-quality gDNA material prior to library preparation. Sequencing was performed using the 2 × 151-bp paired-end format on the MiSeq platform at Axil Scientific Pte. Ltd. (Singapore). The library was prepared using a Nextera XT library preparation kit (Illumina) following the manufacturer’s instructions. The library (8 pM) was sequenced using a 300-cycle MiSeq reagent 2 microkit (Illumina) with an average sequencing coverage of 40×, a read length of 2 × 150-bp paired ends, and a total read length of 3.7 Mb. The quality and quantity of the reads were determined using Agilent TapeStation 4200, PicoGreen, and quantitative PCR (qPCR). Default parameters were used for all software unless otherwise specified. An initial annotation was made using the NCBI Prokaryotic Genome Annotation Pipeline using the best-placed reference protein set and GeneMarkS-2+. The draft genome was found to be 5.6 Mb long, with a GC content of 36.5%. A total of 50 contigs were obtained, with 48 containing protein-encoding genes and a total of 5,823 putative genes.

The sequence was examined using antiSMASH 4.0.2 (7, 8). The bacterial database was set as the reference depository, and gene cluster BLAST comparative analysis and the secondary metabolism gene families (smCOGs) function were selected with strict detection parameters that only detect well-defined clusters containing all required parts. A total of 13 biosynthetic gene clusters were identified. There is one cluster encoding the biosynthetic pathway for siderophores, four nonribosomal peptide synthetase (NRPS) gene clusters, one terpene, four bacteriocins, one sactipeptide, one arylpolyene, and one cluster that had no predicted compound family. Two predicted pathways, a siderophore and a sactipeptide gene cluster, showed high homology (100% similarity) to known biosynthetic gene clusters for the biosynthesis of petrobactin (9–11) and thurincin H (12), respectively.

The *Bacillus* sp. strain 007/AIA-02/001 genome sequence provides information on a potential anti-quorum sensing compound producer (5) isolated from *C. singaporensis*. The number of unique secondary metabolite biosynthetic gene clusters suggests that the bacterial strain may be a promising source for new quorum sensing inhibitory molecules, as supported by the mass spectrometry (MS) metabolomics profile of its organic extracts (5).

### Data availability.

The raw sequence data have been deposited and made publicly available at DDBJ/ENA/GenBank under the accession number PRJNA516514. The whole-genome shotgun project has been deposited at DDBJ/ENA/GenBank under the accession number SEIY00000000. The version described in this paper is the first version.
